# Therapeutic Potential of Human Umbilical Cord-Derived Mesenchymal Stem Cells in Recovering From Murine Pulmonary Emphysema Under Cigarette Smoke Exposure

**DOI:** 10.3389/fmed.2021.713824

**Published:** 2021-09-27

**Authors:** Xiao-Yue Chen, Yi-Ying Chen, Willie Lin, Chien-Han Chen, Yu-Chieh Wen, Ta-Chih Hsiao, Hsiu-Chu Chou, Kian Fan Chung, Hsiao-Chi Chuang

**Affiliations:** ^1^Graduate Institute of Medical Sciences, College of Medicine, Taipei Medical University, Taipei, Taiwan; ^2^School of Respiratory Therapy, College of Medicine, Taipei Medical University, Taipei, Taiwan; ^3^Meridigen Biotech Co., Ltd., Taipei, Taiwan; ^4^Graduate Institute of Environmental Engineering, National Taiwan University, Taipei, Taiwan; ^5^Department of Anatomy and Cell Biology, School of Medicine, College of Medicine, Taipei Medical University, Taipei, Taiwan; ^6^National Heart and Lung Institute, Imperial College London, London, United Kingdom; ^7^Division of Pulmonary Medicine, Department of Internal Medicine, Shuang Ho Hospital, Taipei Medical University, New Taipei City, Taiwan; ^8^Cell Physiology and Molecular Image Research Center, Wan Fang Hospital, Taipei Medical University, Taipei, Taiwan

**Keywords:** cigarette smoke, COPD, emphysema, inflammation, stem cell

## Abstract

Human umbilical cord-derived mesenchymal stem cells (hUC-MSCs) were shown to have potential for immunoregulation and tissue repair. The objective of this study was to investigate the effects of hUC-MSCs on emphysema in chronic obstructive pulmonary disease (COPD). The C57BL/6JNarl mice were exposed to cigarette smoke (CS) for 4 months followed by administration of hUC-MSCs at 3 × 10^6^ (low dose), 1 × 10^7^ (medium dose), and 3 × 10^7^ cells/kg body weight (high dose). The hUC-MSCs caused significant decreases in emphysema severity by measuring the mean linear intercept (MLI) and destructive index (DI). A decrease in neutrophils (%) and an increase in lymphocytes (%) in bronchoalveolar lavage fluid (BALF) were observed in emphysematous mice after hUC-MSC treatment. Lung levels of interleukin (IL)-1β, C-X-C motif chemokine ligand 1 (CXCL1)/keratinocyte chemoattractant (KC), and matrix metalloproteinase (MMP)-12 significantly decreased after hUC-MSC administration. Significant reductions in tumor necrosis factor (TNF)-α, IL-1β, and IL-17A in serum occurred after hUC-MSC administration. Notably, the cell viability of lung fibroblasts improved with hUC-MSCs after being treated with CS extract (CSE). Furthermore, the hUC-MSCs-conditioned medium (hUC-MSCs-CM) restored the contractile force, and increased messenger RNA expressions of elastin and fibronectin by lung fibroblasts. In conclusion, hUC-MSCs reduced inflammatory responses and emphysema severity in CS-induced emphysematous mice.

## Introduction

Chronic obstructive pulmonary disease (COPD) is currently one of the world's highest causes of mortality and ranks fifth worldwide in terms of disease burden (1–3). About 80–90% of COPD patients are related to cigarette smoking (4). A previous study found that exposure to cigarette smoke (CS) for 12 weeks induced emphysematous lung lesions in rats (5). This irreversible alveolar destruction and emphysematous changes due to CS exposure resulted in higher mortality and difficulties in treating COPD.

Mesenchymal stem cells (SCs; MSCs), multipotent SCs, have high self-renewal and differentiation capacities (6). Recent studies demonstrated immunoregulatory functions of MSCs in treating graft vs. host disease (7, 8). Also, tissue-repair actions of MSCs through a paracrine mechanism were explored (9, 10). Notably, most intravenously (i.v.) administered MSCs were localized in the lungs (11). Recruitment of MSCs to the lungs provides new insights that MSCs may have greater paracrine effects in the lungs. Therefore, the effects of MSCs on lung disease treatment were recently noted (12, 13).

Human umbilical cord-derived (hUC)-MSCs have a higher differential capacity, lower immunogenicity, and less age-related dysfunction compared to adult SCs (14). Other advantages of hUC-MSCs are that there are fewer ethical issues associated with them and they can be non-invasively collected (15). Anti-inflammatory effects of hUC-MSCs were found in an acute lung injury mouse model (16). Moreover, it was demonstrated that hUC-MSCs prevented bleomycin-induced lung fibrosis *in vivo* (17).

Lung fibroblasts were shown to have an important role in repairing damaged lung tissues after CS exposure (18). However, a previous study found a decrease in the proliferation of lung fibroblasts in COPD (19). Recently, the senescence-associated secretory phenotype of lung fibroblasts was found in CS-induced emphysema (20). Consequently, the loss of the ability to repair alveoli due to CS was mainly because of lung fibroblast dysfunction (21, 22). MSCs were shown to mediate the proliferation and increase the pro-collagen expression of lung fibroblasts (23).

Despite the efficacy of MSCs in ameliorating acute lung damage, few studies have investigated the effects of hUC-MSCs on chronic CS-induced emphysema. The objective of this study was to investigate the therapeutic efficacy of hUC-MSCs in emphysema.

## Materials and Methods

### Animals

The animal study was approved by the Animal and Ethics Review Committee of the Laboratory Animal Center at Taipei Medical University, Taipei, Taiwan (IACUC: LAC-2017-0231). Male C57BL/6JNarl mice (8 weeks, 20–25 g, *n* = 8–10 per group) were obtained from the National Laboratory Animal Center (Taipei, Taiwan). Mice were housed in plastic cages and supplied with Lab Diet 5001 (PMI Nutrition International, St. Louis, MO, USA) and water *ad libitum*. A light/dark cycle of 12 h/12 h was maintained. The room temperature was set to 22 ± 2°C, and relative humidity to 55 ± 10%.

### CS-Induced Emphysema

**Figure 1:**
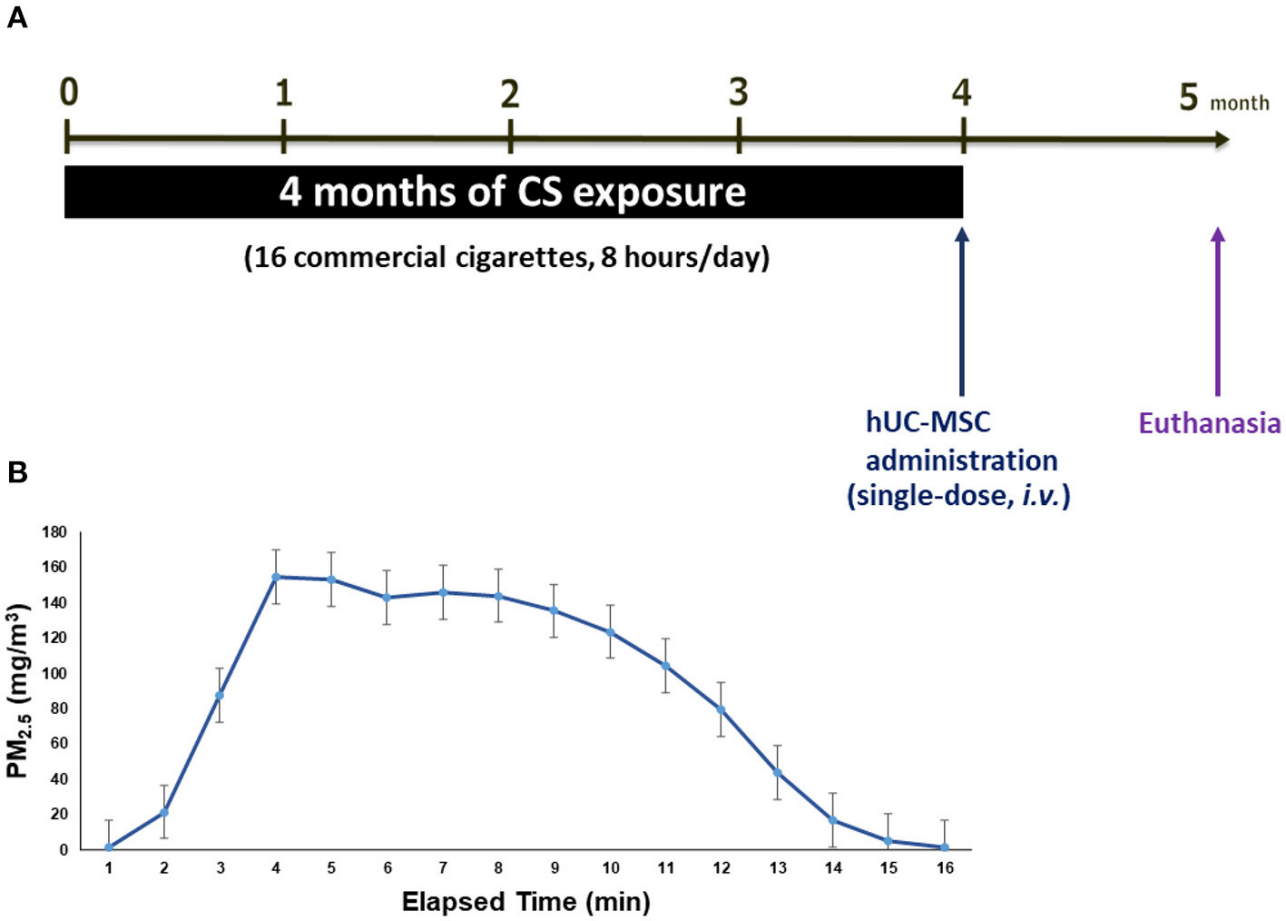
**(A)** Schematic mice model of cigarette smoke (CS)-induced emphysema. **(B)** The distribution of particulate matter with an aerodynamic diameter of <2.5 μm (PM_2.5_) mass concentration in the whole-body exposure system (mean ± SD). Mice (8 weeks old, 20–25 g, *n* = 8–10 per group) were exposed to CS for 4 months and received (i.v.) a single dose of human umbilical cord-derived mesenchymal stem cells (hUC-MSCs) after CS exposure (CS + MSC-L: hUC-MSCs at 3 × 10^6^ cells/kg body weight (BW) for low-dose, CS + MSC-M: 1 × 10^7^ cells/kg BW for medium-dose, and CS + MSC-H: 3 × 10^7^ cells/kg BW for high-dose).

An emphysema mouse model was established by whole-body exposure to CS for 4 months. Details of the CS exposure system were previously reported (24). Briefly, the system consisted of a CS generator, a whole-body exposure chamber (TECNIPLAST, VA, Italy), and a particulate matter (PM) monitor. A side-stream was introduced into the whole-body exposure chamber at a flow rate of 15 L/min. There were 16 commercial cigarettes (Longlife, Taipei, Taiwan; 11 mg of tar and 0.9 mg of nicotine) combusted for 8 h/day and 5 days/week for 4 months (Figure 1A). The mass concentration of PM of <2.5 μm in aerodynamic diameter (PM_2.5_) was monitored using a DustTrak monitor (8530, TSI, Shoreview, MN, USA). Figure 1B shows the distribution of the PM_2.5_ mass concentration during CS exposure. The average PM_2.5_ mass concentration was 90.5 ± 40.6 mg/m^3^ during the first 15 min. It reached a maximum level of about 154.3 ± 58.2 mg/m^3^ after 4 min of cigarette combustion, and then the mass concentration declined to the baseline level after 16 min. Simultaneously, mice exposed to CS-free high-efficiency particulate air (HEPA)-filtered room air (RA) served as the control group.

### hUC-MSC Preparation and Characterization

Details of hUC-MSC preparation were previously reported (24). Briefly, umbilical cords were aseptically harvested and digested with collagenase (SERVA, Heidelberg, Germany) at 37°C. The cell pellets were expanded in α-minimal essential medium (α-MEM, Invitrogen, Carlsbad, CA, USA), and cultured in an incubator with 5% CO_2_ at 37°C for 3 days. hUC-MSCs were characterized using flow cytometry (BD Stemflow™ hMSC Analysis Kit; BD Biosciences, San Jose, CA, USA) to detect expressions of cluster of differentiation (CD) markers (CD11b, CD19, CD34, CD44, CD45, CD73, CD90, and CD105) and human leukocyte antigen–antigen D related (HLA-DR). As presented in Supplementary Table 1, hUC-MSCs exhibited positive expressions of SC-specific surface markers (CD44, CD73, CD90, and CD105) and negative expressions of CD11b, CD19, CD34, CD45, and HLA-DR, which followed International Society for Cellular Therapy Guidelines (25). hUC-MSCs were prepared in clinical-grade normal saline supplemented with 2% clinical-grade human serum albumin and 16.7% clinical grade CS10. This study was approved by the Ethics Committee of the National Cheng Kung University Hospital Institutional Review Board (Tainan, Taiwan; IRB no.: A-BR-104-045). All subjects received written and oral informed consent before inclusion. All study processes were conducted following the approved study protocol.

### hUC-MSC Administration and Sample Collection

The experimental design is shown in Figure 1A. After 4 months of CS exposure, emphysematous mice were randomly divided into four groups: sham control (CS), low-dose group (CS + MSC-L), medium-dose group (CS + MSC-M), and high-dose group (CS + MSC-H). Mice were intravenously (i.v.) administrated a single-dose of hUC-MSCs at 3 × 10^6^ cells/kg body weight (BW) for CS + MSC-L, 1 × 10^7^ cells/kg BW for CS + MSC-M, and 3 × 10^7^ cells/kg BW for CS + MSC-H. The administered dose of hUC-MSCs was referenced to our previous reports (24, 26). The control and CS sham groups were i.v. administrated the same volume of vehicle. BW was measured once a week before and after hUC-MSC administration. Mice were euthanized 4 weeks after hUC-MSC administration. Bronchoalveolar lavage fluid (BALF), lung tissues, and serum were collected. For histological analyses, lung samples were inflated with 10% (m/v) paraformaldehyde in phosphate-buffered saline (PBS) at a pressure of 21 cm H_2_O.

### Emphysema Evaluation

Lung tissues were embedded in paraffin and sectioned into slices for staining with hematoxylin and eosin (H&E). The mean linear intercept (MLI) and destructive index (DI) were used to evaluate the presence of emphysema. The MLI was assessed by counting the number of the alveolar walls intercepted in the grid lines, according to previously described methods (27, 28). The DI for microscopic lung lesions was previously reported (27, 28). Emphysematous defects or intramural parenchyma in at least two intersections of alveoli were considered alveolar destruction.

### Hematology

BALF was centrifuged at 1,500 rpm for 10 min at 4°C. Cell pellets were resuspended in PBS. Numbers of neutrophils, lymphocytes, monocytes, and eosinophils were quantified by a hematology analyzer (ProCyte Dx, IDEXX Laboratories, Westbrook, ME, USA). Data are expressed as percentages (%) of total cell counts.

### Proteins Extracted From Lung Tissues

Lysis buffer was prepared from 490 μL of lysis reagent (Sigma-Aldrich, St. Louis, MO, USA) containing 5 μL of a protease inhibitor (Geno Technology, St. Louis, MO, USA) and 5 μL of ethylenediaminetetraacetic acid. Lung tissues were homogenized in lysis buffer using a homogenizer (Minilys® personal homogenizer, Bertin, Rockville, MD, USA).

### Cytometric Bead Array (CBA) and Enzyme-Linked Immunosorbent Assay (ELISA)

A CBA (BD Biosciences, San Jose, CA, USA) was used to quantify levels of tumor necrosis factor (TNF)-α, interleukin (IL)-1β, chemokine (C-X-C motif) ligand 1/keratinocyte chemoattractant (CXCL1/KC), and IL-17A in BALF, lung, and serum samples. Matrix metalloproteinase (MMP)-12 was determined in lung samples by an ELISA (Cloud-Clone, Katy, TX, USA). Quantification of these markers in lung samples was normalized to the total protein. All measurements were undertaken in accordance with the manufacturers' instructions.

### Human Lung Fibroblasts

Human lung fibroblasts (MRC-5 cells) were obtained from the Food Industry Research and Development Institute (FIRDI, Hsinchu, Taiwan) and cultured in T75 flasks with Eagle's minimum essential medium (EMEM, Lonza Group, Basel, Switzerland) supplemented with 10% fetal bovine serum (FBS), 2 mM L-glutamine, 0.1 mM non-essential amino acids, and 1 mM sodium pyruvate.

### hUC-MSCs-Conditioned Medium (CM) Preparation

To collect hUC-MSCs-CM, hUC-MSCs (1.2 × 10^6^ cells) were cultured in T75 flasks with 15 mL of hUC-MSC culture medium for 24 h. After being washed with PBS, the culture medium was replaced with 10 mL of α-MEM basal medium (Invitrogen, Carlsbad, CA, USA) and incubated for 48 h. The subsequent serum-free culture medium was collected and served as hUC-MSCs-CM.

### CS Extract (CSE)

CSE was prepared from the combustion of three cigarettes (Marlboro, Philip Morris, VA, USA) by impinging onto 30 mL of α-MEM (Invitrogen) with a firm filter. The cigarette contained 10 mg of tar and 0.8 mg of nicotine. Fresh CSE was collected to serve as 100% CSE and immediately used for cell experiments.

### Cell Viability of Human Lung Fibroblasts by hUC-MSCs After CSE Exposure

MRC-5 cells were treated with 8% CSE for 24 h and then indirectly cocultured with hUC-MSCs for another 48 h. Cell viability of MRC-5 cells was determined by a cell counting kit-8 (Merck, Darmstadt, Germany).

### Cell Contractile Force and Elastin and Fibronectin of Human Lung Fibroblasts by hUC-MSCs-CM After CSE Exposure

MRC-5 cells (2 × 10^5^/cells) seeded in six-well plates were treated with 8% CSE for 24 h. After CSE exposure, cells were cultured in hUC-MSCs-CM for 24 h. The cell contractile force was measured using a collagen-based cell contraction assay kit (CellBiolabs, San Diego, CA, USA). Messenger (m)RNA expressions of elastin and fibronectin were analyzed by a quantitative polymerase chain reaction (qPCR), according to the manufacturer's instructions.

### Statistical Analysis

Data are presented as the mean ± standard deviation (SD). Multiple groups were compared by an analysis of variance (ANOVA) with Tukey's *post-hoc* test. An unpaired *t*-test was used for comparisons between continuous variables. All analyses were performed using GraphPad vers. 6 (San Diego, CA, USA). *p* < 0.05 was considered statistically significant.

## Results

### hUC-MSCs Mitigated Emphysema Severity

**Figure 2:**
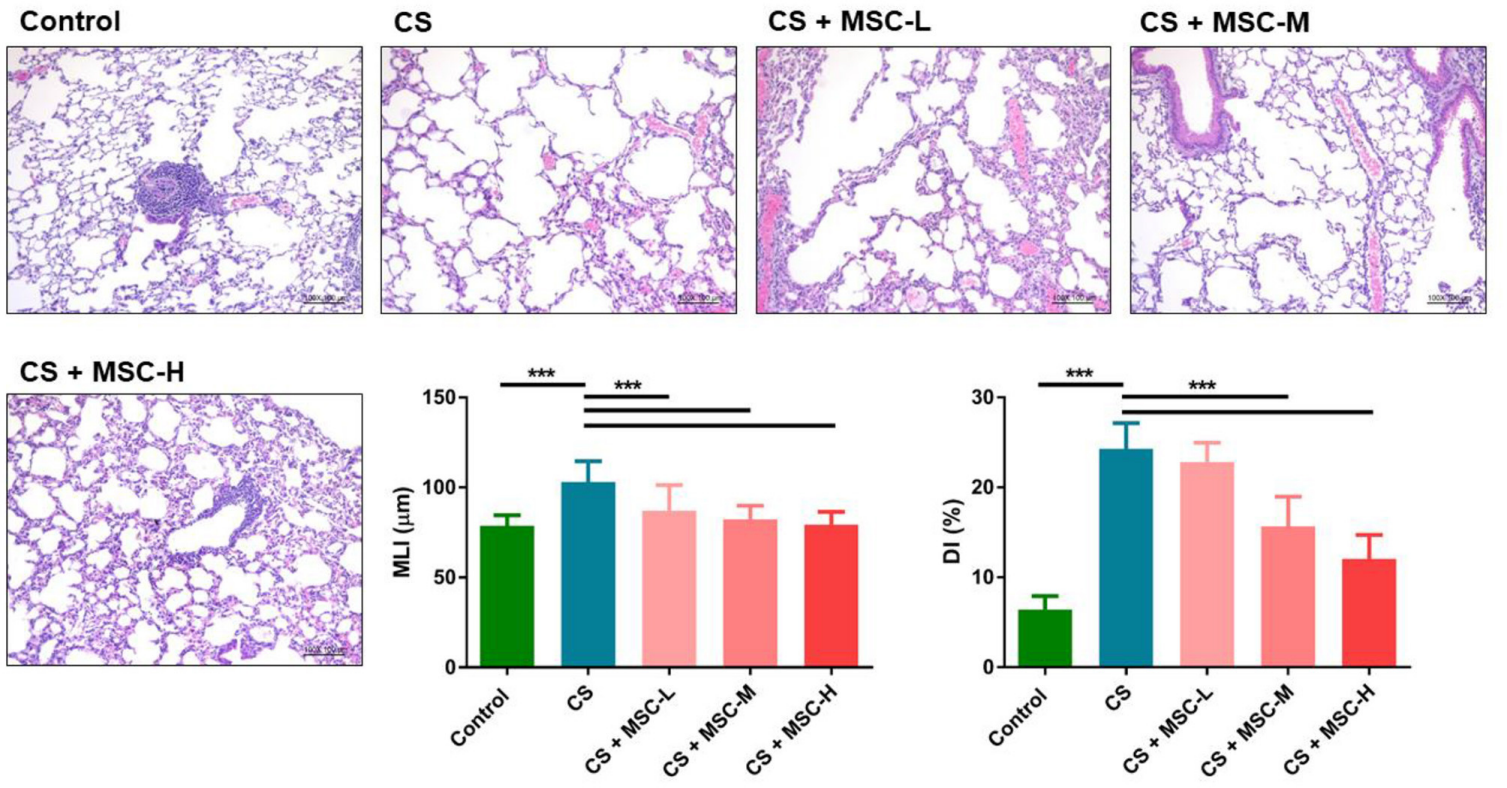
Repair of alveolar structures by human umbilical cord-derived mesenchymal stem cells (hUC-MSCs) in a mice model of cigarette smoke (CS)-induced emphysema. Lung tissue sections were stained with hematoxylin and eosin (H&E). Lung lesions were quantified by measuring the mean linear intercept (MLI) and destructive index (DI). Significant reductions of the MLI and DI (%) were observed by hUC-MSC administration after CS exposure for 4 months. Results were determined by a one-way ANOVA with Tukey's test. *n* = 8–10 per group. ^***^p < 0.001.

Results of the histological analysis are shown in Figure 2. A significant decrease in the MLI by hUC-MSCs was observed compared to the CS group (low-dose: 87.08 ± 14.20, medium-dose: 82.34 ± 7.50, and high-dose MSCs: 79.32 ± 7.14 vs. the CS group: 103.10 ± 11.52 μm, *p* < 0.001). Furthermore, the DI (%) significantly decreased after hUC-MSC administration (medium-dose: 15.67 ± 3.30% and high-dose MSCs: 12.05 ± 2.65% vs. the CS group: 24.30 ± 2.85%, *p* < 0.001).

### Reduction of Lung Infiltration by hUC-MSCs

**Figure 3:**
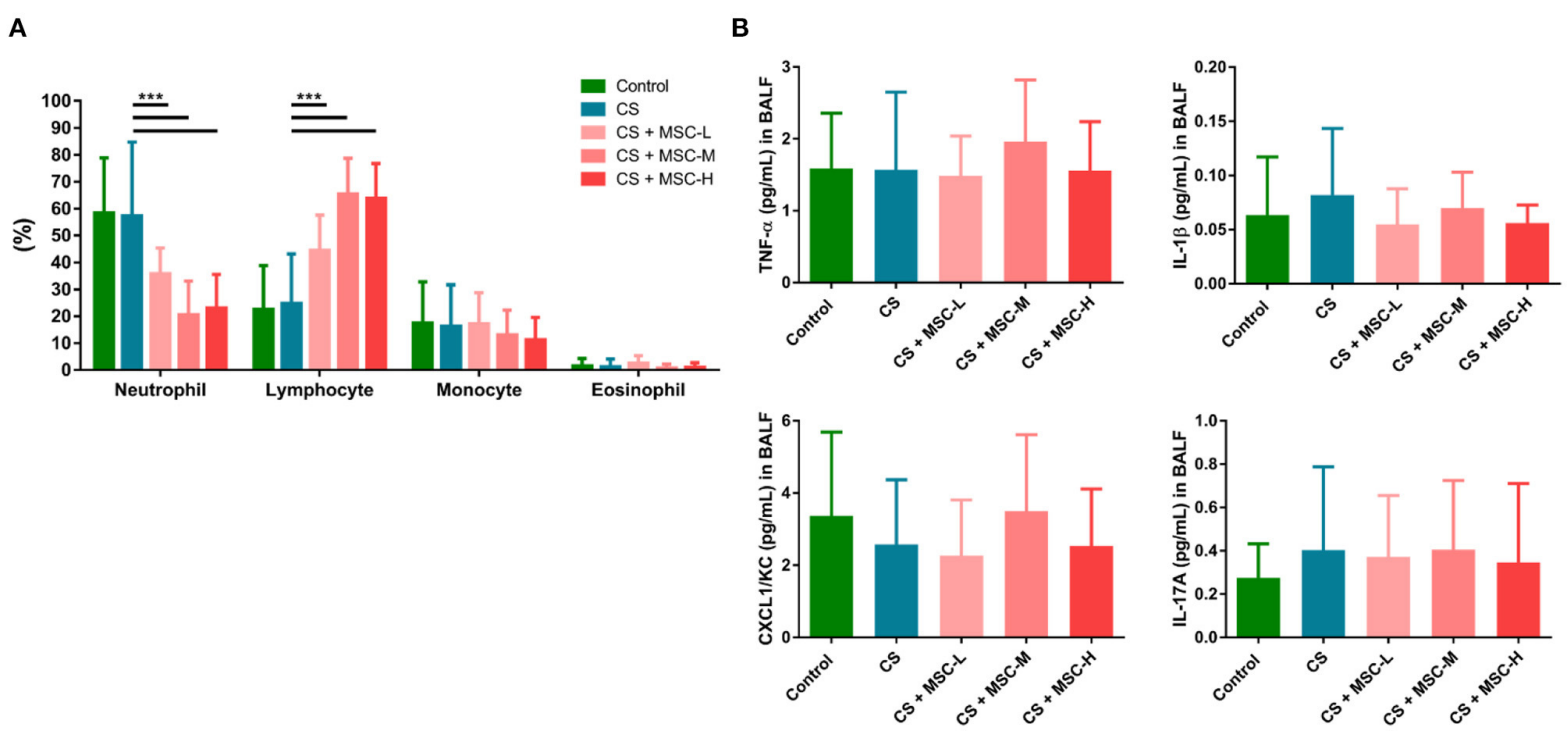
**(A)** The human umbilical cord-derived mesenchymal stem cells (hUC-MSCs) reduced neutrophils and increased lymphocytes in bronchoalveolar lavage fluid (BALF) of mice. **(B)** Regulation of cytokine production (tumor necrosis factor (TNF)-α, interleukin (IL)-1β, C-X-C motif chemokine ligand 1 (CXCL1)/keratinocyte chemoattractant (KC), and IL-17A) by hUC-MSCs in BALF. A significant decrease in neutrophils (%) was observed by hUC-MSC administration, whereas lymphocytes (%) increased after hUC-MSC administration. There was no significant difference in cytokine production in BALF after hUC-MSC administration. The results were determined by a one-way ANOVA with Tukey's test. *n* = 8–10 per group. ^***^p < 0.001.

As shown in Figure 3A, a significant decrease in the percentage of neutrophils was observed in the hUC-MSC group compared to the CS group (low-dose: 35.83 ± 9.50%, medium-dose: 20.64 ± 12.44%, and high-dose MSCs: 23.05 ± 12.54% vs. the CS group: 57.29 ± 27.45%, *p* < 0.001). In contrast, lymphocytes (%) significantly increased after hUC-MSC administration compared to the CS group (low-dose: 44.47 ± 13.17%, medium-dose: 65.44 ± 13.29%, and high-dose MSCs: 63.73 ± 13.08% vs. the CS group: 24.77 ± 18.41%, *p* < 0.001). There was no statistical difference in monocytes (%) or eosinophils (%) among the groups. Also, we observed no statistical difference in TNF-α, IL-1β, CXCL1/KC, or IL-17A in BALF after hUC-MSC administration (Figure 3B).

### hUC-MSCs Decreased Levels of IL-1β, CXCL1/KC, and MMP-12 in the Lungs

**Figure 4:**
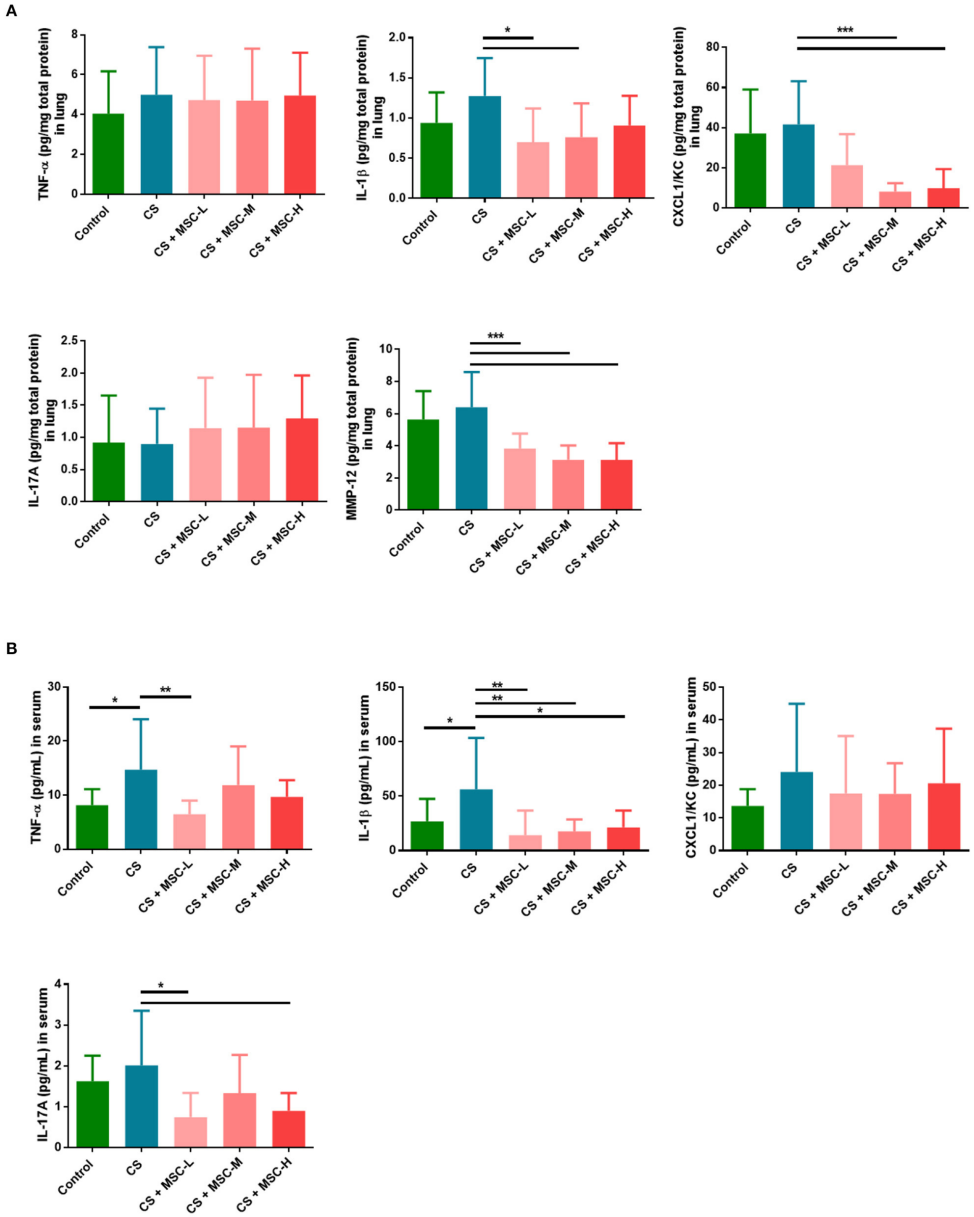
**(A)** Human umbilical cord-derived mesenchymal stem cells (hUC-MSCs) downregulated interleukin (IL)-1β, C-X-C chemokine ligand 1 (CXCL1)/keratinocyte chemoattractant (KC), and matrix metalloproteinase (MMP)-12 in lung lysates. **(B)** hUC-MSCs decreased systemic cytokine production (tumor necrosis factor (TNF)-α, IL-1β, and IL-17A) in serum. Mice lungs were homogenized, and then lung lysates and facial blood of mice were measured by a CBA or ELISA. IL-1β, CXCL1/KC, and MMP-12 in the lungs of mice were significantly reduced by hUC-MSCs. Significant decreases in TNF-α, IL-1β, and IL-17A in the serum of mice by hUC-MSCs were seen, and data were determined by a one-way ANOVA with Tukey's test. *n* = 8–10 per group. ^*^p < 0.05, ^**^p < 0.01, ^***^p < 0.001.

Levels of IL-1β (low-dose: 0.70 ± 0.42 and medium-dose MSCs: 0.76 ± 0.42 vs. the CS group: 1.28 ± 0.47 pg/mg, *p* < 0.05) and CXCL1/KC (medium-dose: 8.20 ± 4.14 and high-dose MSCs: 9.92 ± 9.47 vs. CS group: 41.61 ± 21.56 pg/mg, *p* < 0.001) in lung lysates significantly decreased after hUC-MSC administration compared to the CS group (Figure 4A). Also, we found that MMP-12 in lungs of mice was significantly reduced by hUC-MSCs (low-dose: 3.83 ± 0.92, medium-dose: 3.14 ± 0.89, and high-dose MSCs: 3.13 ± 1.03 vs. the CS group: 6.40 ± 2.20 pg/mg, *p* < 0.001). There was no significant change in TNF-α or IL-17A levels among all groups.

### hUC-MSCs Reduced Levels of TNF-α, IL-1β, and IL-17A in Serum

TNF-α, IL-1β, CXCL1/KC, and IL-17A levels in serum of mice were examined (Figure 4B). hUC-MSCs significantly reduced levels of TNF-α (low-dose MSCs: 6.49 ± 2.48 vs. the CS group: 14.71 ± 9.34 pg/mL, *p* < 0.01), IL-1β (low-dose: 14.16 ± 22.48, medium-dose: 17.69 ± 10.86, and high-dose MSCs: 21.4 ± 15.27 vs. the CS group: 56.31 ± 47.24 pg/mL, *p* < 0.05), and IL-17A (low-dose: 0.75 ± 0.59 and high-dose MSCs: 0.90 ± 0.44 vs. the CS group: 2.02 ± 1.34 pg/mL, *p* < 0.05) compared to the CS group. No significant reduction in CXCL1/KC was found when compared among all groups.

### Proliferation of Lung Fibroblasts by hUC-MSCs

**Figure 5:**
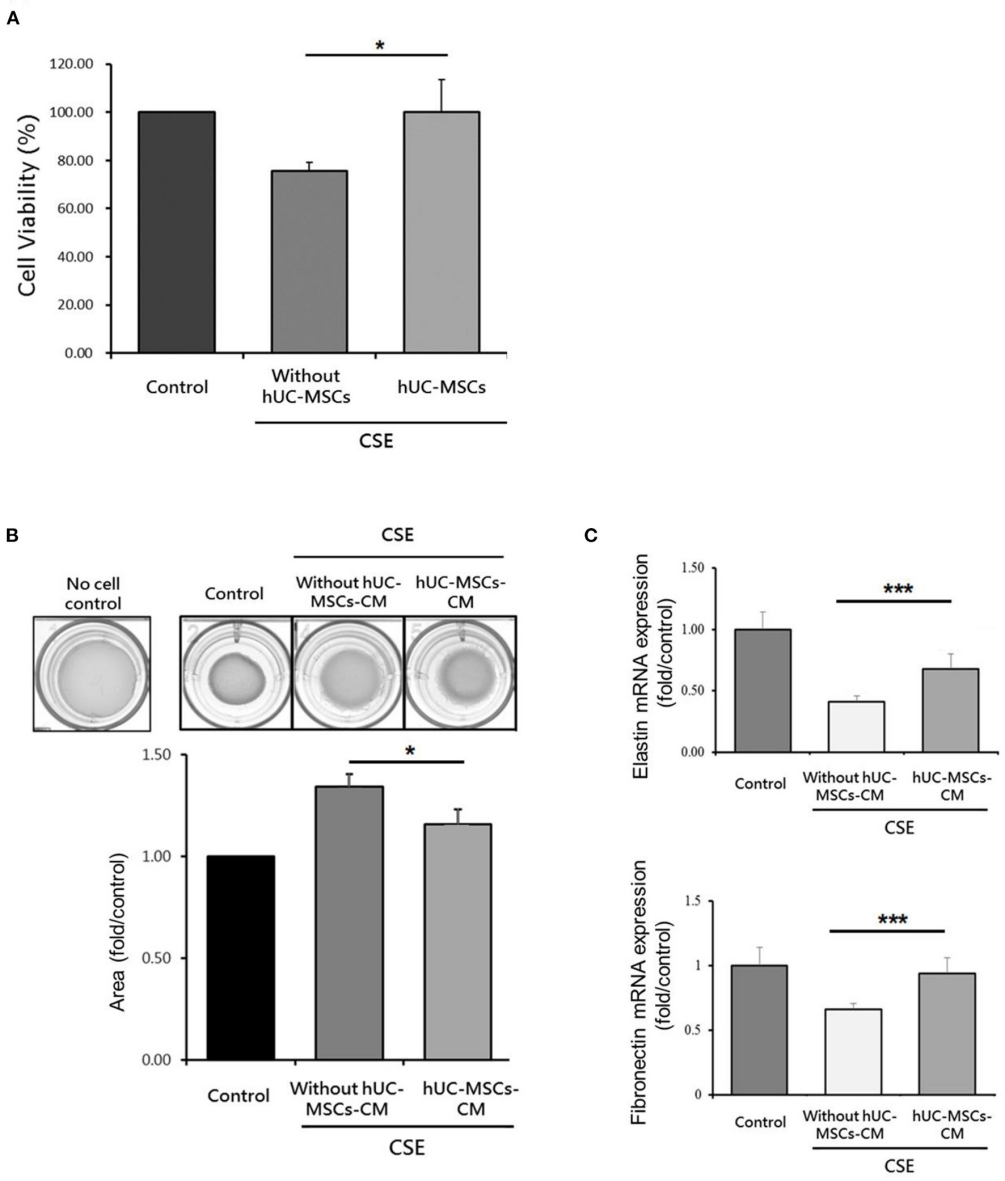
**(A)** Increased cell viability of human lung fibroblasts by human umbilical cord-derived mesenchymal stem cells (hUC-MSCs) after cigarette smoke extract (CSE) exposure. **(B)** Restoration of the contractile force of lung fibroblasts by hUC-MSCs-conditioned medium (hUC-MSCs-CM). **(C)** hUC-MSCs-CM increased mRNA expressions of elastin and fibronectin in lung fibroblasts after CSE treatment. A transwell coculture system was used to determine cell viability of human lung fibroblasts (MRC5 cell line). A significant increase in cell viability (%) of MRC5 cells by hUC-MSCs was observed after CSE treatment for 24 h. MRC-5 cells were treated by CSE for 24 h and then received hUC-MSCs-CM for 24 h afterward. The contractile force was evaluated by a collagen-based cell contraction assay. The mRNA expressions of elastin and fibronectin were quantified by a qPCR. Results were examined by an unpaired *t*-test. Four independent experiments in each group. ^*^p < 0.05, ^***^p < 0.001.

As shown in Figure 5A, the cell viability of MRC-5 cells significantly increased by hUC-MSCs after CSE treatment compared to the CSE group (*p* < 0.05). The contractile force of MRC-5 cells as determined by the collagen gel surface area was significantly reduced by hUC-MSCs-CM treatment compared to the CSE group (*p* < 0.05; Figure 5B). A significant increase in mRNA expressions of elastin and fibronectin were observed by hUC-MSCs-CM treatment compared to the CSE group (*p* < 0.001; Figure 5C).

## Discussion

MSCs were shown to have the potential for immunomodulation and tissue regeneration in different diseases (29–31). We observed that hUC-MSCs decreased the emphysema severity and reduced lung and systemic inflammatory infiltration in mice with CS-induced emphysema. Moreover, we observed that hUC-MSCs increased the proliferation of lung fibroblasts after CSE exposure. hUC-MSCs may ameliorate emphysematous lung lesions in COPD.

Mice were exposed to CS for 4 months at an average mass concentration of 90.5 ± 40.6 mg/m^3^ PM_2.5_ to induce development of emphysema in the present study. The CS-exposure system in this study was described previously (24). Previous reports also showed that CS exposure for 12–14 weeks was able to induce an emphysema model (32–35). During the CS exposure, the mice were significantly decreased in body weight and a significant increase in the serum level of TNF-α as compared to the control before hUC-MSC administration (Supplementary Figures 1A,B). After 4 months of exposure to CS, we observed significantly increased emphysema severity (MLI and DI) and elevation of pro-inflammatory factors (TNF-α and IL-1β) in serum without a significant change in BW (Supplementary Figure 2). The observation suggests that a mouse model of CS-induced emphysema was successfully established in the present study. However, it is worth to note that the mice were euthanized 4 weeks after the CS exposure. This may result in the decrease of inflammatory responses in the CS group.

The lungs are an important organ for accumulation of hUC-MSCs after their administration (36–38). Lung inflammatory infiltration was mitigated by hUC-MSCs in emphysematous mice. First, neutrophils were significantly reduced in BALF by hUC-MSCs. Previous studies showed that neutrophils or polymorphonuclear cells decreased in BALF by MSC administration after CS exposure for 7~16 weeks *in vivo* (35, 39). Pulmonary neutrophil activation by CS is reported to be associated with pro-inflammatory activation and alveolar destruction by releasing neutrophil elastase in COPD (40–42). Therefore, hUC-MSC administration is able to reduce increasing levels of neutrophilic inflammation. Next, we observed that lymphocytes significantly increased in BALF after hUC-MSC administration. Another study showed that intranasal delivery of MSCs slightly increased lymphocytes in BALF of mice compared to the intraperitoneal route in mice with CS-induced emphysema (43). Those results pointed out that different routes and timing of MSC administration could have distinct effects on regulating immune cell populations. MSCs transiently activate T cells to preserve the antiapoptotic function (44). For example, higher lymphocyte counts were more efficient in activating MSCs in the treatment of graft vs. host disease (45). A previous study showed that that hUC-MSCs recruited the regulatory T cells in the damaged lung (46). Together, hUC-MSCs could regulate lung neutrophil infiltration and lymphocyte activation in emphysematous mice. However, more experiments should be conducted in the future to support this.

We observed that inflammatory responses of the lungs, including IL-1β, CXCL1/KC, and MMP-12, by CS decreased after administration of hUC-MSCs. Consistent with a previous study, pro-inflammatory cytokines (TNF-α, IL-1β, and monocyte chemoattractant protein-1) and proteases (MMP-9 and−12) in the lungs of rats decreased by MSC administration after CS exposure for 11 weeks (47). In addition, we found that serum levels of TNF-α, IL-1β, and IL-17A significantly decreased by hUC-MSC administration after CS exposure. TNF-α, IL-1β, and IL-17A were shown to be key mediators in recruiting neutrophils to the lungs after CS exposure (48–53). Previous studies have found that the MMP-12 liberated the neutrophil chemoattractants (e.g., TNF-α) from the macrophage, which recruited the neutrophils and released the elastase that contributes to the lung damage (41, 54–56). It was hypothesized that MSCs may protect the pulmonary matrix structure by reducing MMP and elastase productions in alveolar macrophages and neutrophils, respectively (41, 57–59). Our results showed that decreases in serum levels of neutrophil chemotactic factors, including TNF-α, IL-1β, and IL-17A by hUC-MSCs may possibly be associated with the reduction in neutrophils in the BALF of mice after CS exposure.

The emphysema severity was significantly decreased by hUC-MSCs in emphysematous mice based on the MLI and DI results. Previous studies showed a decrease in emphysematous lesions in the lungs of mice due to bone marrow (BM)-MSCs (60, 61). Other studies found that MSCs induced neutrophil apoptosis and decreased protease secretions resulting in reduced severity of COPD (62–64). In our study, one explanation for the mitigation of the emphysema was decreased levels of pro-inflammatory factors in the lungs (IL-1β and CXCL1/KC) and circulation (TNF-α, IL-1β, and IL-17A) by hUC-MSCs which may associate with the reduction of the neutrophil infiltration in emphysematous mice. In addition, the decrease in protease secretion (MMP-12) by hUC-MSCs contributed to reducing alveolar destruction. Our results suggest that hUC-MSCs may ameliorate alveolar destruction in mice after CS-induced emphysema. However, the underlying mechanisms should be investigated in the future.

Fibroblasts play an important role in regulating COPD severity. We observed that the cell viability of lung fibroblasts increased by hUC-MSC administration after CSE exposure. In addition, hUC-MSCs-CM restored collagen's contractile force in lung fibroblasts after treatment with the CSE. MSCs-conditioned medium (MSCs-CM) was compatible with MSCs in attenuating inflammation in bronchopulmonary dysplasia (65). A previous study showed that MSCs-CM induced lung fibroblast proliferation and restored their repair function after CSE exposure (66). Consistent with previous findings, our results showed that mRNA expressions of elastin and fibronectin by lung fibroblasts significantly increased after treatment with hUC-MSCs-CM compared to the group treated with CSE alone. Collectively, these results suggested that paracrine factors secreted by hUC-MSCs to lung fibroblasts may be partly involved in the alveolar repair process after CS exposure.

There are a few limitations in this study. We observed an increase in lymphocytes in BALF of mice due to hUC-MSC administration. The different subgroups of lymphocytes, including regulatory T cells, were not determined in our study. In addition, interactions of hUC-MSCs with lymphocytes are not fully understood. The pulmonary function and the underlying mechanism of the hUC-MSCs in COPD will be determined in the future. Moreover, the adverse effects of fibroblasts by hUC-MSCs *in vivo* are still unclear, which should be evaluated in future work.

## Conclusions

In conclusion, hUC-MSCs reduced the emphysema severity and inflammatory responses in mice with CS-induced emphysema. hUC-MSCs increased the proliferation of fibroblasts after CSE exposure. hUC-MSCs may mitigate COPD in mice after CS exposure.

## Data Availability Statement

The original contributions presented in the study are included in the article/Supplementary Material, further inquiries can be directed to the corresponding author/s.

## Ethics Statement

The studies involving human participants were reviewed and approved by Ethics Committee of the National Cheng Kung University Hospital Institutional Review Board (Tainan, Taiwan; IRB no.: A-BR-104-045). The patients/participants provided their written informed consent to participate in this study. The animal study was reviewed and approved by Animal and Ethics Review Committee of the Laboratory Animal Center, Taipei Medical University, Taipei, Taiwan (IACUC: LAC-2017-0231).

## Author Contributions

H-CC and X-YC contributed to interpretation of the data and completion of the manuscript. H-CC, WL, and KC contributed substantially to the concept, design, interpretation of the data, and completion of the study and manuscript. Y-YC and C-HC contributed substantially to the completion of the study. T-CH contributed to the establishment of the cigarette smoke generation system and particle measurement. All authors contributed to critically revising the manuscript for important intellectual content and read and approved the final manuscript.

## Funding

This study was supported by a Grant from Meridigen Biotech Co., Ltd., Taipei, Taiwan (2017-TR-VIV-001). The fund was used for designing the study, collecting, analyzing, interpreting the data, and writing the manuscript.

## Conflict of Interest

WL, C-HC, and Y-CW are employed by Meridigen Biotech Co., Ltd. The remaining authors declare that the research was conducted in the absence of any commercial or financial relationships that could be construed as a potential conflict of interest.

## Publisher's Note

All claims expressed in this article are solely those of the authors and do not necessarily represent those of their affiliated organizations, or those of the publisher, the editors and the reviewers. Any product that may be evaluated in this article, or claim that may be made by its manufacturer, is not guaranteed or endorsed by the publisher.
