# Supplementary material for: Therapeutic Potential of Human Umbilical Cord-Derived Mesenchymal Stem Cells in Recovering From Murine Pulmonary Emphysema Under Cigarette Smoke Exposure
Source: Front Med (Lausanne). 2021 Sep 27;8:713824. doi: 10.3389/fmed.2021.713824 (PMC8502916; doi:10.3389/fmed.2021.713824)

Supplementary Information

Therapeutic Potential of Human Umbilical Cord-Derived Mesenchymal Stem Cells in Recovering from Murine Pulmonary Emphysema under Cigarette Smoke Exposure

Xiao-Yue Chen[1,2] (MSc), Yi-Ying Chen[2] (MSc), Willie Lin[3] (PhD), Chien-Han Chen[3] (MSc), Yu-Chieh Wen[3] (MSc), Ta-Chih Hsiao[4] (PhD), Hsiu-Chu Chou[5] (PhD), Kian Fan Chung (MD, PhD)[6], Hsiao-Chi Chuang[2,7,8]* (PhD)

[1]Graduate Institute of Medical Sciences, College of Medicine, Taipei Medical University, Taipei, Taiwan

[2]School of Respiratory Therapy, College of Medicine, Taipei Medical University, Taipei, Taiwan

[3]Meridigen Biotech Co., Ltd., Taipei, Taiwan

[4]Graduate Institute of Environmental Engineering, National Taiwan University, Taipei, Taiwan

[5]Department of Anatomy and Cell Biology, School of Medicine, College of Medicine, Taipei Medical University, Taipei, Taiwan

[6]National Heart and Lung Institute, Imperial College London, London, UK

[7]Division of Pulmonary Medicine, Department of Internal Medicine, Shuang Ho Hospital, Taipei Medical University, New Taipei City, Taiwan

[8]Cell Physiology and Molecular Image Research Center, Wan Fang Hospital, Taipei Medical University, Taipei, Taiwan

Supplementary Information

Table S1. Expression of cell surface markers on hUC-MSCs.

The cell surface markers on hUC-MSCs were determined by flow cytometry. The hUC-MSCs positively expressed the CD44, CD73, CD90, and CD105, whereas negative expression of CD11b, CD19, CD34, CD45, and HLA-DR.

Positive Markers	Positive cells (%)
CD44	100
CD73	100
CD90	98.9
CD105	99.8
Negative Markers	
CD11b/CD19/CD34/CD45/HLA-DR	0.41

Supplementary Information

Figure S1. The change of body weight and serum level of TNF-α with CS exposure before hUC-MSC administration.

(A) The body weight was significantly decreased during the CS exposure. (B) A significant increase in TNF-α in the serum of mice after CS exposure for 4 months. The results were analyzed by an unpaired *t*-test. n = 8~10 per group. *$p < 0.05$, **$p < 0.01$, ****$p < 0.0001$.

(A) **(B)**

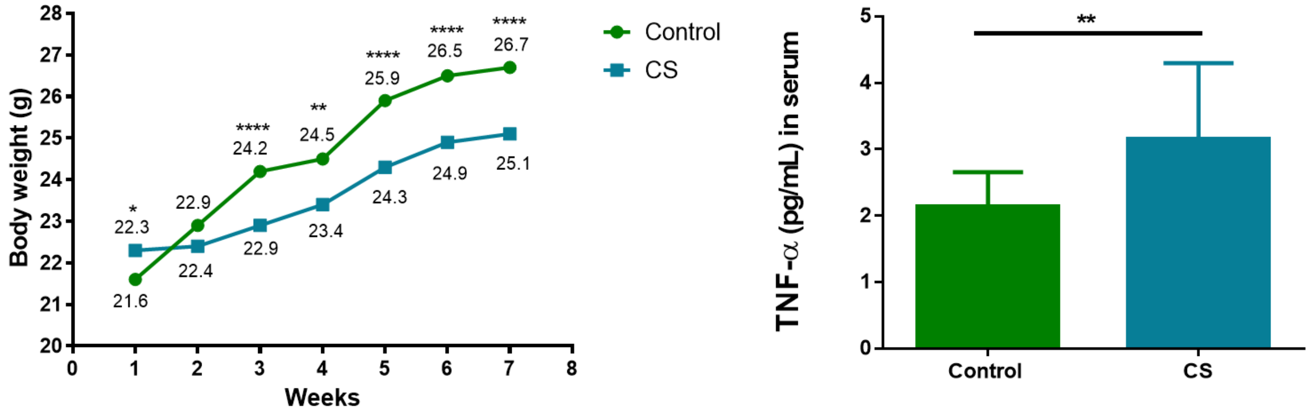

Supplementary Information

Figure S2. The body weight difference before and after 4-week hUC-MSC administration. n = 8~10 per group.

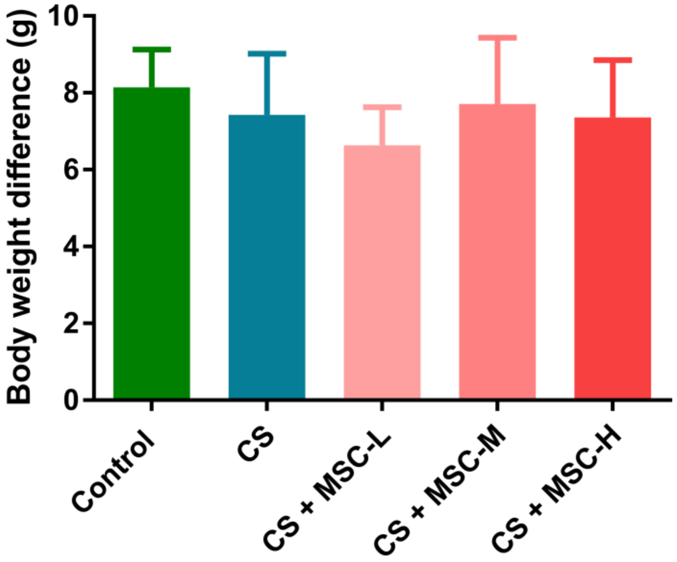

Supplement: Supplementary file 1 [file Data_Sheet_1.pdf]
